# Feasibility of a Smartphone website to support antenatal Perineal massage in pregnant women

**DOI:** 10.1186/s12884-017-1536-9

**Published:** 2017-10-16

**Authors:** Shoko Takeuchi, Shigeko Horiuchi

**Affiliations:** 1 Yokohama City University, 3-9 Fukuura, Kanazawa-ku, Yokohama, Kanagawa 236-0004 Japan; 2 St. Luke’s International University, 10-1 Akashicho, Chuo-ku, Tokyo, 104-0004 Japan; 3 St. Luke’s Maternity Care Home, Tokyo, Japan

**Keywords:** Perineum, Massage, Pregnant women, Smartphone, Website, Feasibility

## Abstract

**Background:**

In Japan, 85% of pregnant women do not practice antenatal perineal massage. Therefore, we developed a smartphone website to support the practice of antenatal perineal massage. The purpose of the present study was to evaluate the feasibility of our smartphone website.

**Methods:**

Pregnant women were recruited at five hospitals or clinics in Tokyo, Japan. Participants assigned to the smartphone website group (*n* = 74) were asked to register on the smartphone website. After completing registration, they could login and use all the contents of the website. After giving birth, participants completed a 5-item questionnaire evaluating the acceptability of the smartphone website. Participants assigned to the leaflet group (*n* = 71) received a leaflet on antenatal perineal massage and completed a similar 4-item questionnaire evaluating the leaflet. Data were collected from April 2014 to November 2014. Data analysis was performed using chi-square and t-tests to analyze responses to close-ended questions, and content analysis was conducted to analyze responses of open-ended questions.

**Results:**

In the smartphone website group, 9 women (12.2%) did not register on the smartphone website. Approximately 80% of the women who responded indicated that the smartphone site was easy to understand and useful for practicing antenatal perineal massage. In the smartphone website group, the reply rate for reporting the frequency of massage was 43.6%. Although the ratings and frequency at which the material was accessed tended to be higher in the smartphone website group than in the leaflet group, there were no significant differences.

**Conclusions:**

Most pregnant women in the smartphone website group provided a favorable evaluation for the smartphone website. However, some participants had suggestions for improvement, which need to be incorporated in a revised version of the website. Therefore, the present study’s results demonstrate the feasibility of a smartphone website to support the practice of antenatal perineal massage, and they may aid in the development of similar web-based educational material for pregnant women.

**Trial registration:**

This trial was registered with the UMIN Clinical Trials Registry (UMIN000013979) on May 16, 2014.

## Background

Perineal trauma is common following vaginal delivery and includes perineal lacerations and episiotomy. Selective episiotomy is associated with less severe perineal or vaginal trauma and fewer healing complications compared to that for routine use [1]. On the other hand, episiotomy has long-term effects, such as urinary incontinence [2], coital pain [3], and unease in subsequent deliveries [4]. Despite this, the episiotomy rate is 30–100% for primiparous women in Japan, with episiotomies being routinely performed at some hospitals [5]. Thus, methods to reduce the incidence of perineal trauma, including episiotomy, are needed.

Antenatal perineal massage is one method that appears to reduce the incidence of perineal trauma following childbirth. A recent systematic review reported that antenatal perineal massage in primiparous women was associated with a significantly reduced incidence of trauma requiring suturing and episiotomy [6]. However, 51.6% of Japanese urban medical facilities do not provide education regarding antenatal perineal massage (Shimizu k: How do you provide intrapartum care to low-risk women?, unpublished). Furthermore, 85% of Japanese pregnant women do not practice antenatal perineal massage [7]; therefore, it is inferred that many pregnant women also do not receive education regarding this type of massage from their midwives.

In developed countries around the world, the Internet has become an important educational tool. Numerous studies have demonstrated the effectiveness of web-based education in terms of improved knowledge, awareness, and self-efficacy, and a systematic review regarding Internet-based interventions demonstrated their effectiveness in behavioral change [8]. In addition, smartphone applications and text messages have been recently used as educational tools for pregnant women [9–11]. In Japan, most individuals use the Internet and smartphones, and the rate of individuals accessing the Internet using a smartphone has increased from 31.4% in 2013 to 56.8% in 2015 [12]. Although there are some limitations such as coverage, cost, and data allowance for accessing the Internet, one of the advantages in using a smartphone is that anyone can connect to the Internet at any time and at any place, with ease. Therefore, we developed a smartphone website to support the practice of antenatal perineal massage in pregnant women. The goals of the smartphone website were to encourage pregnant women to recognize the effectiveness of antenatal perineal massage and to continue practicing the massage until childbirth [13]. The contents of the website included five elements: 1) information regarding the effects of antenatal perineal massage, 2) information regarding the massage technique, 3) a message board for support from a peer group, 4) an inquiry form for communication with a professional, and 5) reminders and messages of encouragement. The purpose of the present study was to evaluate the feasibility of using this smartphone website as educational material supporting antenatal perineal massage for pregnant women compared with that of a leaflet (educational information in print).

## Methods

### Study design, setting, and participants

We conducted an evaluation study in an arm of a randomized controlled trial. One hundred sixty-one primiparous pregnant women from five hospitals or clinics in Tokyo, Japan, participated in this randomized controlled trial that evaluated two different educational materials for prenatal perineal massage. The trial methodology has been previously reported in detail [13]. All participants in the present study were primiparous pregnant women who met all of the following inclusion criteria: 1) the progress of the pregnancy was normal; 2) they were able to read and write in Japanese, and 3) they had a smartphone. The exclusion criterion was pregnant women who did not meet the inclusion criteria.

### Data collection

Eligible women were recruited to the randomized, controlled trial when they visited one of the participating sites at 30–33 weeks of gestation. After consenting to the trial, women who were randomly allocated to the smartphone website group received instructions regarding the website and were asked to register on the website as a member. Registration required a username, password, and email address. After receiving an email confirming registration completion, they could logon to the website and use all of the contents. On the website, they could get information regarding the effects of antenatal perineal massage and the massage technique, post a comment on the message board, and complete an inquiry form to communicate with a professional. When participants completed the inquiry form, the researcher received the inquiry and replied to it within 24 h. Information regarding the massage technique included illustrations and pictures to ease comprehension. In addition, the participants received weekly emails, which included a link to an online form for reporting the frequency at which they had practiced the massage during the past week. After participants completed the online form, the email was automatically sent to participants. The email included a different comment according to the number of times they had practiced the perineal massage. For example, a positive comment such as “Excellent!” or “Let’s continue to go like this!” was sent if they practiced the massage more than three times per week. In contrast, if they practiced the massage less than three times per week, an encouraging comment such as “The pain will decrease if you continue the massage.” or “Let’s increase the number of times little by little.” was sent. Women who were allocated to the leaflet group received a leaflet with written information regarding the effects of antenatal perineal massage and the massage technique.

After giving birth, participants in the smartphone website group were asked to complete an online questionnaire to evaluate the smartphone website. The following five items were used to evaluate the acceptability of the smartphone website: 1) comprehensibility of the contents, 2) comprehensibility of the illustrations or pictures, 3) agreement with their expectations, 4) usefulness, and 5) satisfaction with the smartphone website. Items were rated using a four-point Likert scale from 1) I do not agree at all to 4) I agree very much. In addition, an open-ended section was available, in which participants in the smartphone website group were able to freely express their opinions regarding the smartphone website. Participants in the leaflet group completed a written questionnaire to evaluate the leaflet in terms of the following four items: 1) comprehensibility of the contents, 2) agreement with their expectations, 3) usefulness, and 4) satisfaction with the leaflet using a four-point Likert scale. The frequency with which they used the leaflet was also collected in the questionnaire. Data regarding the frequency at which the material was accessed were also collected in both groups. Data were collected from April 2014 to November 2014.

### Statistical analysis

Basic descriptive statistics were performed using SPSS version, 24.0 J (IBM Corp.) for closed-ended items. The open-ended comments providing qualitative data were analyzed using a content analysis following the framework described in Bowen et al. [14], which reported the key areas of focus for feasibility studies to include acceptability, demand, implementation, practicality, adaptation, integration, expansion, and limited efficacy. In addition, chi-square and t-tests were used to evaluate group differences in the ratings and the frequency at which the material was accessed.

## Results

Of 161 women recruited to the randomized, controlled trial, 74 women in the smartphone website group and 71 women in the leaflet group agreed to participate in the present study. Participants’ mean ages were 32.4 years in the smartphone website group (standard deviation [SD] = 4.37 years, range 20–40 years) and 32.6 years in the leaflet group (SD = 4.43 years, range 23–43 years).

### Acceptability of the smartphone website

Twenty-eight women completed the questionnaire evaluating the website. Most women indicated that the website contents were easy to understand (85.7%), the illustrations and pictures were easy to understand (85.7%), their expectations were met (78.6%), the website was useful for practicing perineal massage (82.2%), and they were satisfied with the contents (75.0%). No woman answered “I do not agree at all” for any of the 5 items. In addition, 3 women commented about the accessibility of the smartphone website. Two women felt that the massage was more fun because of the bright design of the website, and the website motivated them to perform the massage. Moreover, a woman said that she was able to maintain her motivation because she was asked weekly to report the frequency at which she had practiced the massage.

### Implementation of the smartphone website

Regarding registration, of 74 women in the smartphone website group, 9 (12.2%) did not register on the website. In addition, 2 women sent emails asking how to register on the smartphone website. After we provided an explanation by email, they were able to register on the website.

Concerning actual use, among 65 women who registered on the smartphone website, the frequency at which the website was accessed averaged 2.2 times per week (SD = 7.4). In addition, 2 women completed the inquiry form with a question on whether baby oil could be used for the perineal massage. No participants posted a comment on the website’s message board. The reply rate for reporting the frequency at which they had practiced the massage during the past week averaged 43.6%.

### Practicality of the smartphone website

Some women reported on the practicality of the smartphone website. Three women commented that the smartphone website provided an opportunity to start perineal massage. Moreover, 4 women reported that they were able to perform perineal massage because the smartphone website provided relevant specific content.

In addition, some suggestions for improvement were reported. Three women more than 40 years old mentioned that a leaflet would be better than the website because their eyes became more tired during pregnancy. Furthermore, 6 women reported that they never became confident in the correctness of their massage technique. Requested improvements included the use of animation and regular updates for the contents.

Regarding the cost to develop the smartphone website, it was included in the set-up charges of the website, development of the contents, server usage, and management; the total cost was 950,000 yen (approximately $8640).

### Adaptation of the smartphone website

Ratings on the comprehensibility of the contents, agreement with expectations, usefulness, and satisfaction were compared between the smartphone website and leaflet groups (48 women in the leaflet group completed the questionnaire). The results are shown in Table 1. A few women in the leaflet group and no women in the smartphone website group answered, “I do not agree at all” for the 4 items. Moreover, the rates at which women responded, “I agree very much” on the items regarding agreement with expectations, usefulness, and satisfaction were larger in the smartphone website group than in the leaflet group. Yet, there were no significant group differences in any of the 4 items.

**Table 1:** Process Outcomes according to Study Groups

	Smartphone website (*n* = 28)	Leaflet (*n* = 48)	*p* value
	n (%)	n (%)	
The contents was easy to understand.			
I agree very much	4 (14.3)	7 (14.5)	.746
I agree	20 (71.4)	33 (68.8)	
I do not agree	4 (14.3)	6 (12.5)	
I do not agree at all	0 (0.0)	2 (4.2)	
The material came up to my expectation.			
I agree very much	3 (10.7)	2 (4.2)	.608
I agree	19 (67.9)	33 (68.8)	
I do not agree	6 (21.4)	12 (24.9)	
I do not agree at all	0 (0.0)	1 (2.1)	
The material was useful for me.			
I agree very much	8 (28.6)	8 (16.6)	.497
I agree	15 (53.6)	32 (66.7)	
I do not agree	5 (17.8)	7 (14.6)	
I do not agree at all	0 (0.0)	1 (2.1)	
I was satisfied with the material.			
I agree very much	5 (17.9)	7 (14.6)	.732
I agree	16 (57.1)	27 (56.3)	
I do not agree	7 (25.0)	12 (24.9)	
I do not agree at all	0 (0.0)	2 (4.2)	

The frequency at which the leaflet was accessed averaged 1.2 times per week (SD = 0.82). The mean frequency at which the smartphone website was accessed tended to be higher than that for the leaflet, but there were no significant group differences.

## Discussion

Almost all the participating women in the smartphone website group indicated that the contents of our smartphone website were easy to understand and useful for practicing perineal massage. The website enabled participants to acquire knowledge regarding perineal massage by including content addressing the effectiveness of the massage and the massage technique. Some participants in the smartphone website group commented that they were able to perform the massage because of the information provided on the smartphone website, although they had been unaware of antenatal perineal massage until they participated in the study. Therefore, the website seems useful for pregnant women who have no prior knowledge of perineal massage. A previous study reported that 34.4% (115/334) of women in Japan indicated that they did not know how to practice antenatal perineal massage [15]. Moreover, a systematic review on Internet use by pregnant women reported that women used the Internet as a source of information on pregnancy [16]. Therefore, our smartphone website may be effective in providing knowledge regarding perineal massage for such women.

In the present study, participants in the smartphone website group received verbal and written instructions on how to register on the smartphone website. However, 12.2% of the women did not register and two additional women had inquiries regarding registration. It may have been difficult to find the icon for the new member registration because the icon size was small. Thus, we plan to make the instructions easier to understand and change the design. Moreover, a username, password, and email address were required to create the account; this allowed only participants of the study in the smartphone website group to use the smartphone website. A previous study exploring young adults’ perspectives on smartphone applications related to health behavior change reported that some individuals preferred password access, whereas others complained about the effort involved in creating accounts and entering passwords [17]. Therefore, the registration method for the smartphone website may have lowered the registration rate, and an easier registration process must be devised.

The message board was included to allow users to share information and provide emotional encouragement by posting messages. It was expected that participants would use the message board and the inquiry form to interact and communicate with peers and professionals, but these tools were rarely used. Previous studies have used numerous strategies to encourage the use of web-based tools, including personalized weekly emails, access to a message board, posting new topics once per week, use of a podcast (an audio file that can be listened to on a computer or mobile media player), use of mobile support communication (Twitter) or a mobile diet monitoring application, and encouraging participants to post at least daily to Twitter [18, 19]. In the current study, such strategies were not used. Although participants in the smartphone website group were encouraged to use the smartphone website and its tools, clearer expectations to engage with the desired research process may be required. Furthermore, the researcher provided only simple instructions regarding the smartphone website. In addition, including frequent updates of interesting and attractive topics on the message board or providing the participants with opportunities to meet and talk with participants living near them and to communicate with participants living far from them through video telephone before using the smartphone website may encourage its use more effectively.

Participants in the smartphone website group provided some requests regarding the information on the antenatal perineal massage technique. The contents of the smartphone website included pictures using a model to explain the massage technique. Although some participants reported that the website was helpful because the contents were concrete, others commented that an animation may improve the comprehensibility. A previous study reported that the rate at which women practiced perineal massage was higher in women who received a video demonstration than in women who received only printed and verbal instructions [20]. Therefore, including an animation, in addition to the still pictures, may improve the understanding of the technique in relation to the movements of the massage.

Additionally, the acceptability ratings and frequency of accessing the smartphone website were compared with those of the leaflet with educational information in print. There were no significant differences between the two educational materials. Therefore, a smartphone website may be adapted as one of the educational materials provided for pregnant women. On the other hand, the cost of the smartphone website is one of the key issues of expanding it. When a website is used as an educational material, not only the development cost but also the running cost, such as the server usage charges and management, is required. Therefore, the cost or cost-effectiveness of the website needs considered.

The present study had several limitations. The data consist of the opinions of a subset of participants who completed the questionnaire. Therefore, the data do not reflect the evaluation of all the participants, including those who did not register on the website. In addition, the sample size was not calculated for the present study, although we calculated the sample size for our previous trial [13], which required 67 women per group. Therefore, the lack of differences found in this study may be due to the small size. Nevertheless, the present study’s results demonstrate the feasibility of a smartphone website to support antenatal perineal massage practice, and they may aid in the development of similar web-based educational material for pregnant women.

## Conclusions

The present study demonstrates the feasibility of a smartphone website as an educational material to support the practice of antenatal perineal massage in pregnant women. In addition, the results clarified the areas requiring improvement. In the future, the website will be revised based on the present results, making it easier to use for many pregnant women. Additionally, the results may provide insights aiding in the development of similar web-based educational material for pregnant women.

## Abbreviations

SD: Standard deviation
